# Supplementary material for: Ethylenediamine loading into a manganese-based metal–organic framework enhances water stability and carbon dioxide uptake of the framework
Source: R Soc Open Sci. 2020 Mar 25;7(3):191934. doi: 10.1098/rsos.191934 (PMC7137959; doi:10.1098/rsos.191934)
Supplement: Electronic Supplementary Information [file rsos191934supp1.pdf]

Ethylenediamine (EDA) loading into a manganese-based MOF enhances water stability and carbon dioxide uptake of the framework.

Aisha Asghar[a], Naseem Iqbal[a]*, LeenaAftab[a] , Tayyaba Noor[b], Benson M. Kariuki[c], Luke Kidwell[c], Timothy L. Easun[c]*

a. United States Pakistan Centre for Advanced Studies in Energy, National University of Sciences and Technology, H-12, Islamabad, Pakistan.
b. School of Chemical and Mechanical Engineering, National University of Sciences and Technology, H-12, Islamabad, Pakistan.
c. School of Chemistry, Cardiff University, Main Building, Park Place, Cardiff CF10 3AT, UK.

SUPPORTING INFORMATION

Table S1: Single crystal XRD data for synthesized Mn-DOBDC sample

**Cell parameters**	
**Formula**	'C_{14} H_{18} Mn N_2 O_8
**Space Group**	I 2/a
**Cell measurement temperature**	298
**a, Å**	9.6916(5)
**b, Å**	11.8690(6)
**c, Å**	15.3430(9)
**Volume, Å^3**	1721.12
**α**	90
**β**	102.788(6)
**γ**	90
**Z**	4
**Absorption coefficient**	0.809
**Goodness of fit**	1.019
**Reflns number total**	2053
**R-factor (%)**	3.69

Table S2. Elemental Composition of Mn-DOBDC & EDA-Mn-DOBDC

**MOF Sample**	**C**	**H**	**N**
Mn-DOBDC	42.31(41.98)	4.53(4.59)	7.05 (6.89)
EDA-Mn-DOBDC	38.70 (39.03)	5.64 (5.81)	15.05 (15.26)

Note: Theoretical values in brackets and calculated values outside brackets

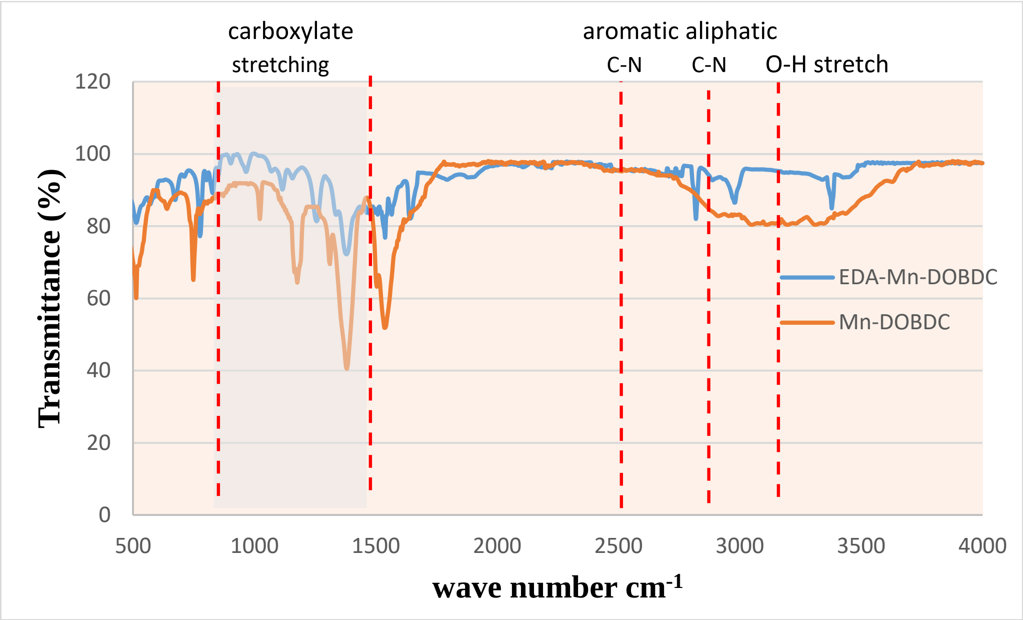

Figure S1. FTIR spectra for Mn-DOBDC (*red*), EDA-Mn-DOBDC *(black*, EDA modified)

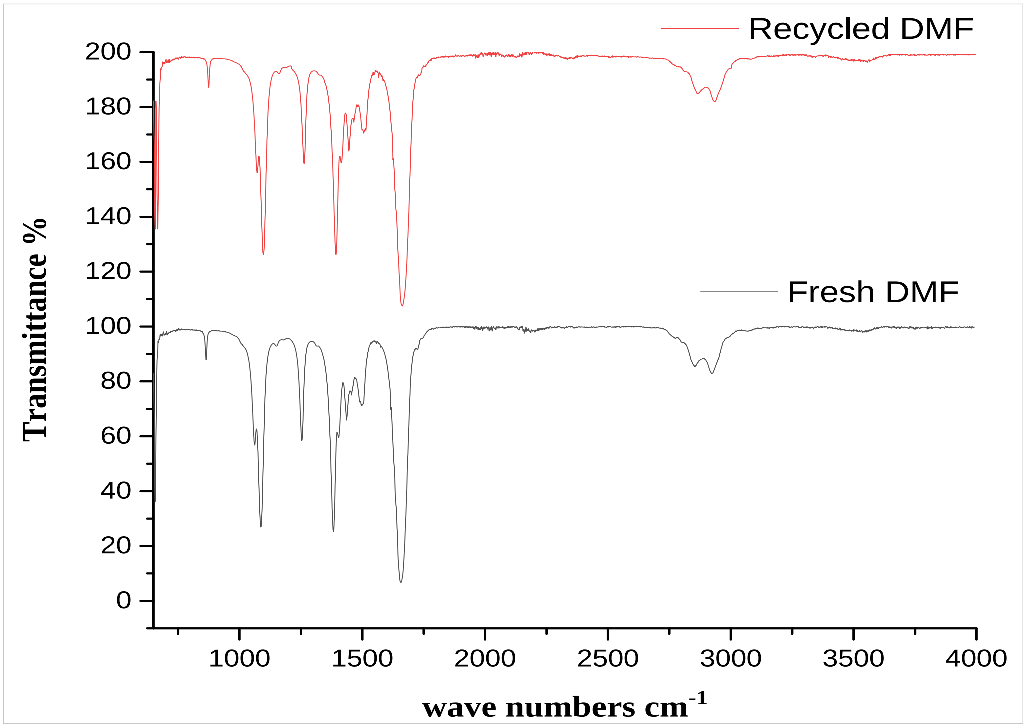

Figure S2. FTIR spectra for fresh and recycled DMF used to make batches I and II of the Mn-DOBDC MOF respectively.

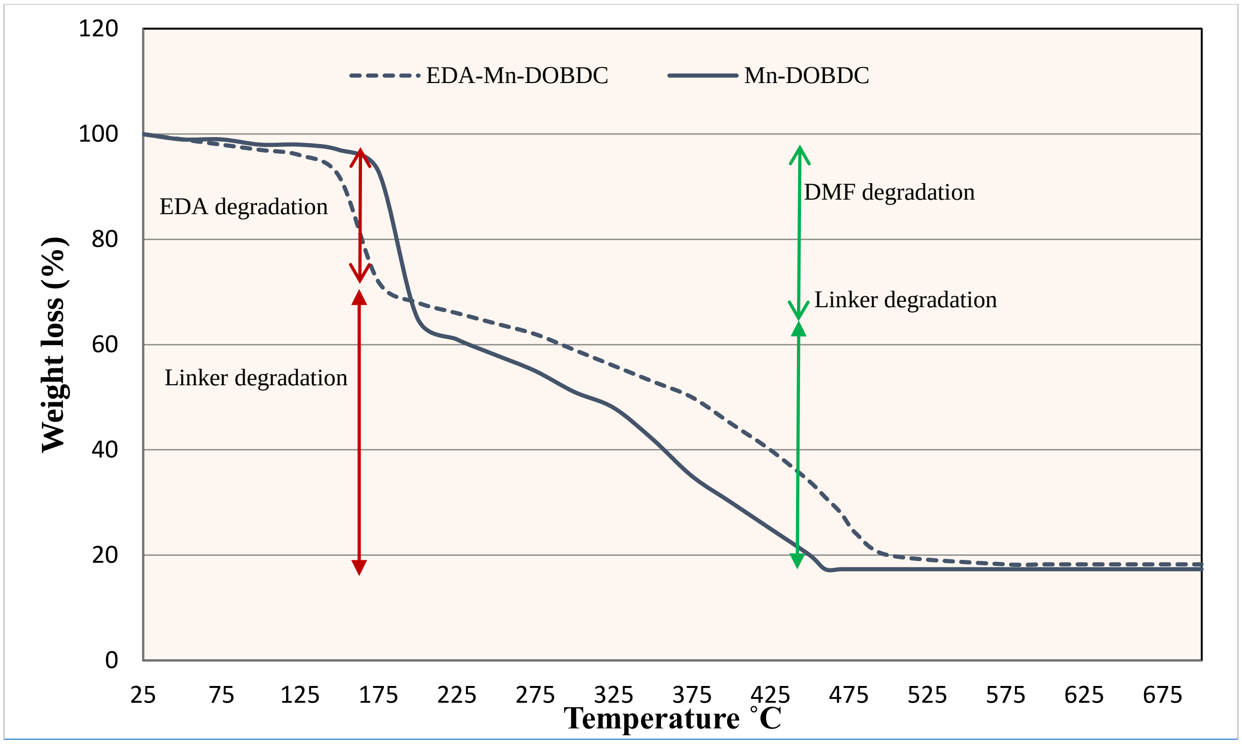

Figure S3. TGA graph for Mn-DOBDC and EDA-Mn-DOBDC; red double headed arrows show degradation of EDA-Mn-DOBDC while green double headed arrows show degradation of Mn-DOBDC.

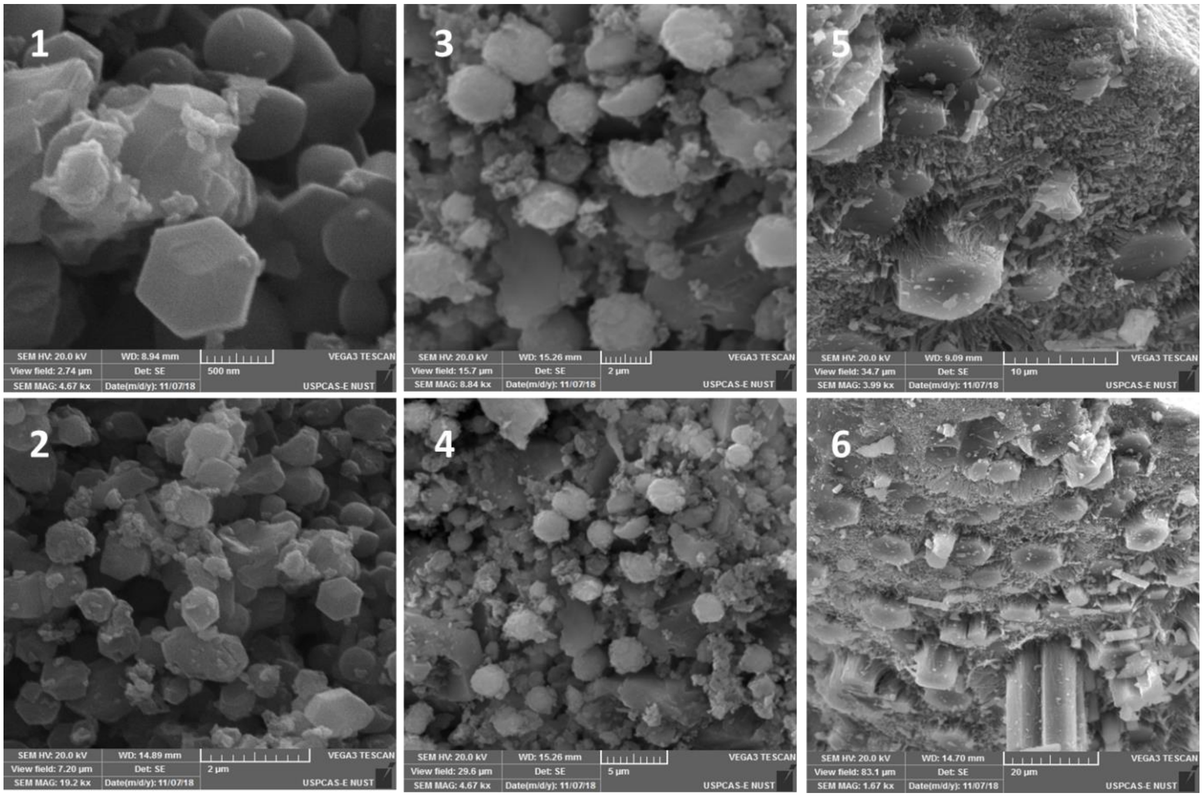

Figure S4. SEM images for Mn-DOBDC batch-I (1 & 2), Mn-DOBDC batch-II (3 & 4) and EDA-Mn-DOBDC (5 & 6).

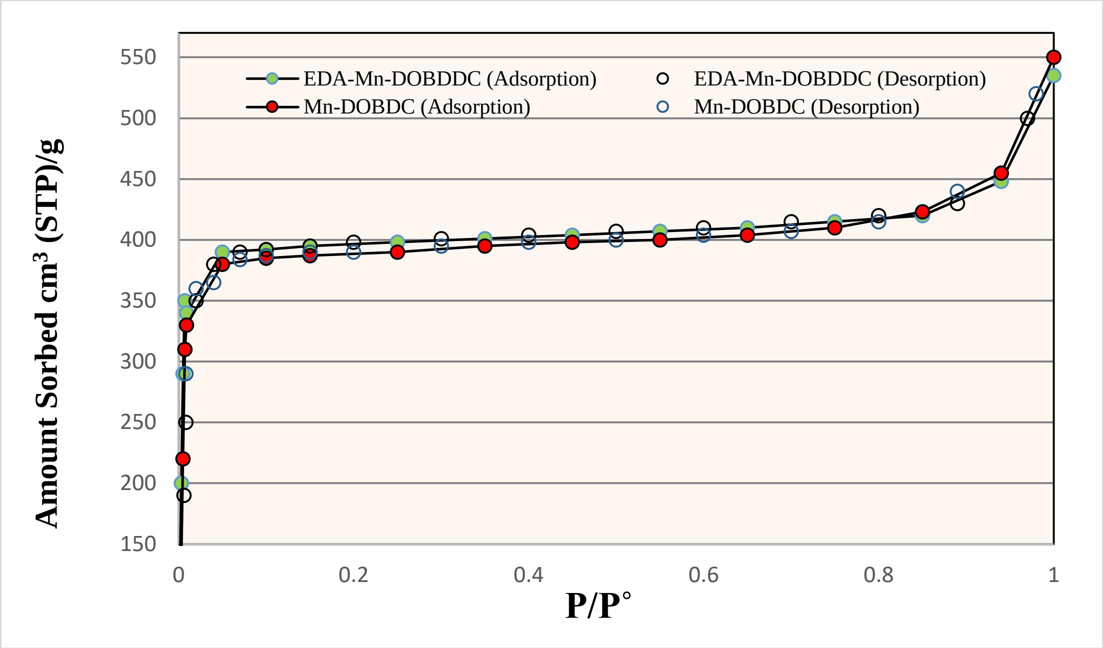

Figure S5. N_2 adsorption-desorption isotherms at 77 K. Adsorption is represented by hollow circles (Red: Mn-DOBDC, Green: EDA-Mn-DOBDC) and desorption is marked by closed circles.

Qst, is the heat Q released in a constant temperature calorimeter when a differential amount of gas is adsorbed at constant pressure. The Van't Hoff isobar equation relates Qst to adsorption isotherms at different temperatures. It is derived from equating the chemical potential of the adsorbed phase and the gas phase, applying the Gibbs Helmholtz relation, and assuming that the vapour phase behaves like an ideal gas. From experimentally obtained isotherms at a constant amount adsorbed and two different temperatures T_1 and T_2, Qst is obtained from the following equation

$$Qst = R\left(\frac{(lnP1 - lnP2)}{(\frac{1}{T1} - \frac{1}{T2})}\right) \qquad \text{(Eq. 1)}$$

where R is the gas constant. Isosteric heat of adsorption for Mn-DOBDC HMTA was calculated using 273 K and 298 K isotherms using the slope of a Van’t Hoff plot against the amount adsorbed. Here Qst decreases with increasing loading indicating strong interaction between the quadrupole moment of carbon dioxide and the adsorbent surface.

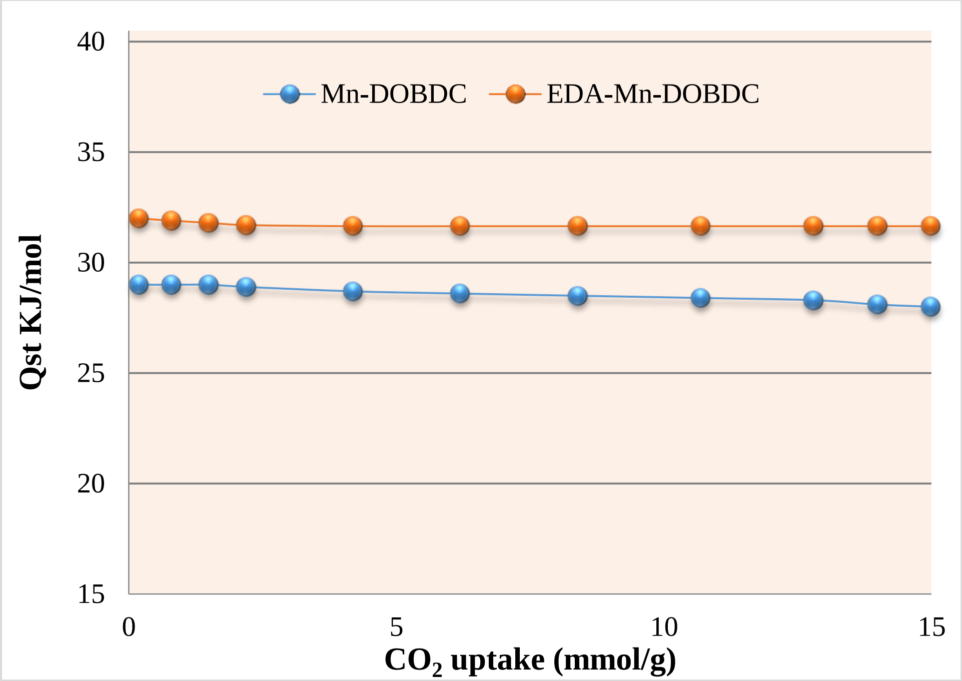

Figure S6. Isosteric heats of CO_2 adsorption onto Mn-DOBDC and EDA-Mn-DOBDC.
